# Proteinases as virulence factors in *Leishmania* spp. infection in mammals

**DOI:** 10.1186/1756-3305-5-160

**Published:** 2012-08-07

**Authors:** Mariana Silva-Almeida, Bernardo Acácio Santini Pereira, Michelle Lopes Ribeiro-Guimarães, Carlos Roberto Alves

**Affiliations:** 1 Laboratório de Biologia Molecular e Doenças Endêmicas, IOC, Fiocruz, Avenida Brasil, 4365 Manguinhos Pavilhão Leônidas Deane – sala 209, CEP: 21040-900, Rio de Janeiro, RJ, Brasil

**Keywords:** *Leishmania* spp., Leishmaniasis, Protease, Proteinase, Lesion

## Abstract

*Leishmania* parasites cause human tegumentary and visceral infections that are commonly referred to as leishmaniasis. Despite the high incidence and prevalence of cases, leishmaniasis has been a neglected disease because it mainly affects developing countries. The data obtained from the analysis of patients’ biological samples and from assays with animal models confirm the involvement of an array of the parasite’s components in its survival inside the mammalian host**.** These components are classified as virulence factors. In this review, we focus on studies that have explored the role of proteinases as virulence factors that promote parasite survival and immune modulation in the mammalian host. Additionally, the direct involvement of proteinases from the host in lesion evolution is analyzed. The gathered data shows that both parasite and host proteinases are involved in the clinical manifestation of leishmaniasis. It is interesting to note that although the majority of the classes of proteinases are present in *Leishmania* spp., only cysteine-proteinases, metalloproteinases and, to a lesser scale, serine-proteinases have been adequately studied. Members from these classes have been implicated in tissue invasion, survival in macrophages and immune modulation by parasites. This review reinforces the importance of the parasite proteinases, which are interesting candidates for new chemo or immunotherapies, in the clinical manifestations of leishmaniasis.

## Review

### Introduction

Leishmaniasis is a vector-borne infection that is present in the Americas, Africa, eastern Europe, western and central Asia, India and Australia [1,2]. The genus *Leishmania* includes protozoan parasites that cause several types of human infections ranging from the visceral form to the tegumentary forms **(**cutaneous, diffuse cutaneous, mucocutaneous and post-kalazar dermal). In addition to humans, animals such as dogs, rodents and marsupials are also susceptible to *Leishmania* infections [3].

Currently, it is estimated that 1–1.5 million new cases of tegumentary leishmaniasis and 0.5 million new cases of the visceral form occur each year [4], and the number of leishmaniasis cases occurring outside of the endemic countries has been increasing due to tourism, military operations and the movement of immigrants from endemic countries [5]. There are two evident morphological phases in the life cycle of these protozoa: (1) elongated promastigotes with visible flagella that inhabit sandfly guts and (2) round-shaped amastigotes without visible flagella that inhabit mammalian cells [6]. During the natural infection, the metacyclic promastigotes are carried by the blood-sucking sandflies that mediate the transmission between mammalian hosts. It has been reported that, in some human cases, the hosts may remain asymptomatic for a long time and thus play an important role in the vector-borne transmission of leishmaniasis in their regions [7].

During the blood meal, the insect vector deposits metacyclic promastigotes in the skin of its host. These promastigotes are the virulent form of *Leishmania* and initiate the infection. The first sign of infection is a small erythema at the site of the sandfly bite that develops after a variable incubation period. The erythema progresses into a papule and then into a nodule that gradually ulcerates over a period ranging from two weeks to six months, eventually becoming the characteristic lesion of localized cutaneous leishmaniasis, as reviewed by [8]. Once in the skin, the parasites are exposed to new microenvironments, such as the extracellular matrix (ECM) of connective tissue, and must interact with a variety of obstacles, including basement membrane proteins, until establishing infection within macrophage phagolysosomes [9,10].

Currently, it is known that the distinct clinical manifestations of leishmaniasis are dependent upon the parasite species and the status of the host’s immune system [10] and result from interactions between host immune factors and parasite components. These manifestations can be divided into three main profiles: (1) an anergic pole, where ulceration is not observed even with a high parasite number in the lesion, characterizes the diffuse cutaneous form; (2) an equilibrated profile characterizes the localized cutaneous form or benign disease, and (3) a hypergenic pole that is observed in mucosal leishmaniasis, in which few parasites are found in the lesions despite the elevated cellular response of the host [11-14].

Some parasite components are characterized as virulence factors that contribute to *Leishmania* pathogenesis and enable the parasite to invade and establish infection in the mammalian host [15]; these virulence factors include glicoinositolphospholipids (GIPLs), lipophosphoglycan (LPG), proteophosphoglycan (PPG) and the 11 kDa kinetoplastid membrane protein (KMP-11). Although the exact impact of these *Leishmania* components on the clinical manifestations observed in mammalian hosts is not yet defined, there is evidence that these components modulate the interactions between *Leishmania* and host immune cells.

For example, GIPLs were reported to help *Leishmania* (*Leishmania*) *major* survive inside macrophages by inhibiting nitric oxide synthase [16] and protein kinase C [17]. Recently, a correlation was shown between the rate of macrophage infection by *Leishmania (Viannia) braziliensis* and the GILP-containing detergent-resistant membrane domains of this parasite [18].

Lipophosphoglycan is a macrophage ligand that is directly involved in the initial stages of the infection. Assays carried out with a mutant strain of *L. (L.) major* deficient in the gene lpg1 (lpg1^-^) showed that the mutant parasites are attenuated for virulence during infections of murine macrophages, despite presenting no major phenotypical changes. These parasites lacked LPG but contained normal levels of related glycoconjugates and GPI-anchored proteins. The lpg1^-^ promastigotes are highly susceptible to the complement system and to the oxidants produced by host cells. In addition, they lose their ability to inhibit phagolysosome fusion. It has also been reported that *L. (L.) major* LPG2 null mutants *(lpg2*^-^) were unable to survive in sandflies or in mammalian host cells. These parasites were more altered than the lpg1^-^ mutants and lacked all phosphoglycans, including LPG and proteophosphoglycans [19-23]*. Leishmania* LPG has been shown to impair the nuclear translocation of NF-κB in monocytes, leading to a subsequent decrease in IL-12 production [24], and can also influence the host’s early immune responses by modulating dendritic cells by inhibiting antigen presentation and promoting an early IL-4 response [25].

Proteophosphoglycans are highly glycosylated polypeptides with O-glycosylations similar to those found in the LPG and acid phosphatase [26,27]. The function of membrane PPGs is not entirely clear, but it is speculated that its long chain that covers the plasma membrane of the parasite could play some role in binding to macrophage receptors. The secretion of modified PPG by parasites within macrophages appears to contribute to maintenance of the parasitophorous vacuole [28]. Additionally, the PPG is also able to activate complement via mannose-binding protein [29].

KMP-11 is a hydrophobic protein that has been described to be associated to LPG [30]. This protein has presented immunoregulatory properties and is able to induce the expression of IL-10 in cells from patients with cutaneous and mucocutaneous leishmaniasis [31]; however, the mechanism through which this effect occurs remains unclear.

### Proteinases of Leishmania as virulence factors

Proteinases are also important virulence factor candidates, as they are enzymes that hydrolyze peptide bonds and thus have the potential to degrade proteins and peptides that participate in a broad range of biological functions, including the infection process (Table 1). Proteinases occur ubiquitously in biological systems and have functions that range from the digestion of proteins for nutritive purposes to the exquisite control of protein function by hydrolyzing a highly specific peptide bond in a protein substrate [32].

**Table 1:** Immunological actions of proteinases on the mammalian immune system that drive clinical manifestations of Leishmaniases

**Proteinase classes**		** *Leishmania* species**	**Activities on mammalian host**	**References**
Cysteine proteinase	**CPA**	*L.* (*L.*) *infantum*	Related to the ability to infect mammalian hosts cells *in vitro;*	[33]
	**CPB**	*L.* (*L.*) *mexicana*	Associated with a Th2 profile in BALB/c mice: inducing lesions; IL-4 and IL-5 production; inhibition of IL-12 production by cleaving NF-κB; inhibition of NO production by cleaving the STAT-1 and AP-1 transcription factors.	[34-42]
			Associated with a Th1 profile in C3HeB/FeJ and C57BL/6 mice: expression of Th1-associated cytokines;	
		*L.* (*L.*) *amazonensis*	Associated with the cleavage of MHC class II gene products in mice;	[43-46]
			Epitopes from CPB COOH-terminal extension modulate infection in BALB/c and CBA mice: induce Th1 or Th2-related cytokines; stimulate CD8+ T lymphocytes;	
			Endogenous CP inhibitors are related with immune modulation	
		*L.* (*L.*) *major*	Associated with a Th1 profile and reduction of IFN-γ expression in C3HeB/FeJ mice;	[37]
		*L. (L.) pifanoi*	Reduces the percentage of infected murine macrophages when parasites are treated with an anti-C-terminal extension antibody	[47]
		*L.* (*L.*) *chagasi*	Associated with Th1 profile in asymptomatic patients and IFN-γ production in cell cultures;	[48]
			Related to Th1 and Th2 profiles in cells from symptomatic human patients and dogs: IFN-γ, IL-4 and IL-10 production in cell cultures;	
	**CPC**	*L.* (*L.*) *mexicana*	Contributes to resisting killing by macrophages	[49-51]
		*L.* (*L.*) *chagasi*	Induces TGF-β expression in human cell culture	[52]
Metallo-proteinases	**M8**	*Leishmania* spp.	Related to the hydrolysis and inactivation of immunoglobulin G; Inactivation of C3b factor to complement C3bi; Adhesion and internalization in macrophages; downregulation of gp63 expression induces Th1 profile in mice; cleave NF-κB and prevent expression of IL-12 and iNOS in mice	[53-56]
		*L.* (*L.*) *major*	Associated with human NK cells proliferation and cleavage CD4 glycoprotein on human T cells in culture.	[57,58]
		*L. (L.) mexicana*	In murine bone marrow macrophages, interferes with signaling cascades and affects transcription factors by cleaving c-Jun, the central component of AP-1, alters signaling through cleavage-activated protein tyrosine phosphatases in murine macrophages	[59]
Serine proteinases	**OPB**	*L.* (*L.*) *donovani*	Allows parasites to infect the murine macrophage	[60,61]
		*L.* (*L.*) *major*	Related to maintenance of murine macrophage infection	[62]

They can be classified, based on their catalytic domains, as serine-, threonine-, aspartyl-, metallo- and cysteine-proteinases [63]. Among these, only the aspartyl-, metallo- and cysteine-proteinase classes have been extensively studied in *Leishmania* species [64-67].

There are several examples of parasite proteinases being involved in pathogenesis and playing roles in parasite invasion and migration through host tissues, degradation of immune related proteins, immune evasion and activation of inflammation [68]. In protozoan parasites, proteinases play key roles in the life cycle transitions, invasion of hosts, migration through tissue barriers, degradation of hemoglobin and other blood proteins, immune evasion, and activation of inflammation in the mammalian host [67,68].

A comparative genomic analysis carried out with the different species of the genus *Leishmania* that have been sequenced revealed that the number of proteinase genes is kept constant among the various species. However, there is a high diversity of proteinases in *Leishmania*, as a survey of multiple databanks reveals that *L.* (*V.*) *braziliensis* alone has at least forty-four cysteine proteinases, twenty-three serine proteinases and ninety-seven metalloproteinase (http://tritrypdb.org, http://blast.ncbi.nlm.nih.gov/). Therefore, due to the broad spectrum of action of *Leishmania* proteinases while the parasite is inside the mammalian host, it is reasonable to propose a correlation between proteinase activity and the clinical manifestation of leishmaniasis.

### Cysteine-proteinases from *Leishmania* as virulence factors

Many studies have identified cysteine proteinases (CPs) as prevalent virulence factors in species that are classified under the *Leishmania (Leishmania) mexicana* complex, especially in the murine infection model used for most of the CP studies. The efficacy of the use of CP inhibitors for infection control can be interpreted as evidence of the importance of these enzymes during the establishment of the infection in the host [69].

Cysteine proteases are enzymes that are known to play critical roles in the pathogenesis of other parasitic protozoa infections, as reviewed by [70], thus their importance as virulence factors and their potential as drug targets and vaccine candidates has been investigated extensively. The most studied CPs in *Leishmania* are designated CPA, CPB and CPC, all of which are papain-like and belong to the same group of CPs, clan CA, that is divided into families, as follows: family C, including cathepsin B-like (e.g., CPC) and cathepsin L-like (e.g., CPA and CPB) enzymes; family C2, including calpain-like enzymes; and others [34,67,69-72].

Several CP genes have been characterized in *Leishmania,* mainly in species of the *L. (L.) mexicana* complex, such as *L*. (*L*.) *mexicana*[73-75], *Leishmania* (*Leishmania*) *pifanoi*[47,76] and *Leishmania* (*Leishmania*) *amazonensis*[77]. The genomic organization and characterization of the cathepsin L-like cysteine proteinases gene cluster from the *Leishmania* (*Leishmania*) *donovani* complex has been previously described [79]. It has also been observed that single nucleotide polymorphisms (SNPs) occurring in CPs, which can vary according to the parasite’s life stage, could be related to clinical characteristics such as a dermotropic rather than a viscerotropic status [79].

Additionally, a high CP activity was observed in extracts of *L. (L.) amazonensis* amastigotes, but promastigotes from the exponential or stationary phases exhibited very low proteolytic activity [77]. In this species of *Leishmania*, a correlation between the levels of CP expression and virulence has been described [80]. In this context, suppression of the CP genes diminished the virulence of *Leishmania* (*Leishmania*) *infantum* in hamsters [81] and of *Leishmania* (*Leishmania*) *chagasi* in human cell cultures [82].

CPs were also shown to play a key role in basic functions and the interactions of *Leishmania (Leishmania*) *tropica* with the host, as parasites treated with CP inhibitors showed reduced viability, growth and pathogenicity [83]. In contrast, CPA was shown to be important in the host-parasite interaction of *L. (L.) infantum* but not to be essential for parasite replication [33].

### CPB as a major virulence factor in species of the *L.* (*L.*) mexicana complex

Proof of the importance of CPBs as a major virulence factor for species of the *L.* (*L.*) *mexicana* complex was obtained in assays of experimental infections in BALB/c mice using genetically modified parasites in which the genes for CPA, CPB and CPC were deleted (*Δcpa, Δcpb* and *Δcpc* strains). These assays showed that the absence of CPA or CPC affected the parasites in a more discrete fashion than the absence of CPB; parasites lacking CPB had a greatly reduced ability to infect and induced fewer lesions than the wild-type strain [34,35].

Other studies analyzed the effects of the reinsertion of *cpb* genes in the *Δcpb* parasite strain; the reinsertion of a single *cpb* gene did not fully restore parasite virulence, but the reinsertion of multiple *cpb* genes (using a cosmid) was able to do so. These data suggest that the multiple copies of *cpb* genes present in the parasites genome produce enzymes with complementary functions [36]. The role of CPB in the progression of infection in distinct murine strains was variable. *L. (L.) mexicana* remained able to continuously induce lesions in BALB/c mice even after the depletion of the *cpb* genes, although it did so at a much lower rate than wild strain parasites, indicating that other virulence factors were still effective. However, in the murine strains C3HeB/FeJ and C57BL/6, the infection with mutant parasites was eventually controlled and the lesions were able to heal [35-37].

Regarding the direct effect of CPB on the parasite’s ability to control host responses, it was observed that *Δcpb L. (L.) mexicana* parasites are unable to promote IL-4 expression during an experimental infection of BALB/c mice. The infected mice were thus able to mount a Th1 response and limit lesion growth. After multiple *cpb* genes were reinserted into the mutant parasites, the capacity to induce IL-4 production was restored along with virulence [36].

It interesting to note that the subversion of immune responses by a specific parasite species may present in different manners depending on the mouse strain used. For example, it has been reported that the virulence of *L.* (*L.*) *mexicana* in the mouse strains C3HeB/FeJ and C57BL/6 is not associated with the capacity to induce IL-4 expression, as in BALB/c mice but is instead due to the inhibition of the expression of Th1-associated cytokines. Studies have shown that animals from these more resistant strains were able to control lesion growth after experimental infection with *Δcpb* parasites but were susceptible to wild strain parasites. In contrast, animals became susceptible to infection by *Δcpb* parasites if the IL12p40 or STAT4 genes were suppressed, suggesting that wild-type parasites inhibit the expression of Th1-related genes [37].

Additionally, experiments using cosmids to insert multiple *L.* (*L.*) *mexicana cpb* genes into *L.* (*L.*) *major* parasites have yielded similar results. After transfection with the *cpb*-containing cosmids, *L.* (*L.*) *major* parasites caused infections with higher parasitic loads and reduced IFN-γ expression in C3HeB/FeJ mice than was observed for infections with the wild-type *L.* (*L.*) *major*, providing more evidence of the influence of CPB on the expression of Th1-related genes [37].

The role of CPB from *L.* (*L.*) *mexicana* in the inhibition of host IL-12 production was also analyzed in macrophages and dendritic cells [38,39]. The role of CPB in IL-12 inhibition was shown using assays with *Δcpb* amastigotes that were less efficient at inhibiting the lipopolysaccharide-related expression of IL-12 than wild-type amastigotes. Supplementary evidence was obtained from the observation that the use of CPB inhibitors in wild-type parasites was similarly able to hinder the ability of amastigotes to inhibit host IL-12 expression [40]. The same study proposed a mechanism through which CPB could inhibit IL-12 expression: the enzyme could be involved in the cleavage of nuclear factor kappa B (NF-κB) and its inhibitors (IκB α and β), thus preventing the expression of interleukins by the host. This IL-12 inhibition could not be observed in assays using *Δcpb* amastigotes or promastigotes, because unlike amastigotes, promastigotes only express low levels of CPB [40].

The influence of CPB from *L.* (*L.*) *mexicana* on the activity of other transcriptional factors was further analyzed in another study that showed that this proteinase affects other transcription factors, such as STAT-1 and AP-1, as well as NF-κB, by impeding their translocation to the nucleus and thus impairing the production of NO that is induced by IFN-γ [41]. Differences in the way that promastigotes and amastigotes modulate NF-κB activity were also observed: while the former cleaves the p65 subunit to a smaller p35 subunit, the latter completely degrades the p65 subunit.

Another effect of CPB on the balance of Th responses occurs through the cleavage of MHC proteins. There is evidence that *L.* (*L.*) *amazonensis* CPB is able to cleave MHC class II proteins inside the parasitophorous vacuole of colonized host cells. Consequently, the host immune response could be partially inhibited or, alternatively, be mediated by other immune components such as the MHC class I gene products [43].

The effects of CPB on the murine host immune system has also been tested by subcutaneously injecting an enzymatically active recombinant CPB protein into the hind paw of BALB/c mice. Even without any actual parasites infecting the mice, there was an increase in IL-4 and IL-5 expression (Th2-related cytokines) and in the levels of circulating IgE. These effects may be partially explained by the ability of CPB to cleave CD23 (low affinity IgE receptor) and CD25 (IL-2 receptor), as a similar assay using an inactive recombinant CPB had no such effects on the murine hosts [42].

### Importance of CPB as a virulence factor in species from other complexes

In spite of the substantial advances in knowledge about CPs from the *L.* (*L.*) *mexicana* complex and, to a lesser degree, of the *L.* (*L.*) *donovani complex*, less is known about CPs from *L.* (*V.*) *braziliensis*, the most important etiological agent of the mucocutaneous form of the disease in the New World. Within this context, our group has been working in an attempt to clarify the biological role of these enzymes. In a recent study, we were able to identify the cellular localization of CPs and their mechanism of anchoring to the parasite plasma membrane [84]. The organization of the *cpb* genes was also determined for this parasite species, along with the subsites of specificity for the recombinant CPB [85].

Most studies about CPB’s activity as an immunomodulator were based on the murine model using species from the *L.* (*L.*) *mexicana* complex; however, an *in vitro* study using cells cultured from dogs and human patients proved that a recombinant CPB from *L.* (*L.*) *chagasi* (rLdccys1) is able to induce cytokine production even in these distinct models. It was shown that rLdccys1 can induce IFN-γ production in cell cultures from asymptomatic patients, IFN-γ, IL-4 and IL-10 in oligosymptomatic patients and IL-4 and IL-10 (at lower levels) in symptomatic patients and dogs [48].

### Immunological effects of the COOH-terminal extension of CPB

In addition to the aforementioned influence of the proteolytic activity of CPB on the host immune system, the COOH-terminal extension (CTE) of CPB has also been reported to influence the interaction of parasites with the host immune system. The CTE is hydrolyzed when CPB is processed to its mature form [86] and is secreted into the extracellular environment [78]. Because the CTE has also been observed inside host cells [65], this polypeptide may interact with and alter the host immune system.

A synthetic peptide based on the CTE of *L. (L.) amazonensis* (PI) was shown to induce the expression of Th2-related cytokines in cells cultured from BALB/c mice, whereas cells from CBA mice exposed to the same peptide expressed cytokines related to both Th1 and Th2 responses. T cell proliferation was also stimulated by the synthetic peptide, especially that of CD8+ T cells. Additionally, when used for *in vivo* assays with infected mice, PI caused increased growth of lesions for BALB/c mice but not for CBA mice [44]. Our group recently conducted a study using *in silico* simulations of the interactions between MHC and CTE-derived epitopes, and the results suggested that these interactions could be related to the production of specific cytokines [45].

The CTE may also play a role in macrophage infections with *L (L.) pifanoi* or *L.* (*L.*) *amazonensis*, as incubation of parasites with anti-CTE antibodies prior to their interaction with macrophages led to a reduction in the number of infected cells. This effect was more dramatic with amastigotes than with promastigotes, and pre-incubation of the parasites with an antibody specific to the *L.* (*L.*) *pifanoi* CPB catalytic domain had no effect in macrophage infection [47].

### Non-CPB cysteine proteinases

Regarding the importance of other non-CPB CPs, studies indicate that CPC also plays a relevant role as a *Leishmania* virulence factor; although amastigotes from a *Δcpc L.* (*L.*) *mexicana* strain were still able to infect macrophages *in vitro* at rates comparable to the wild-type strain [49], these mutant parasites were more susceptible to killing by host cells [50,51]. Additionally, there is evidence that CPC may contribute to some of the immunoregulatory activities of *L.* (*L.*) *chagasi*, such as the parasite’s capacity to induce TGF-β expression in human cell cultures [52].

Regarding CPA, assays based on gene suppression showed that a CPA (*cys1*) from *L.* (*L.*) *infantum* acts as a virulence factor, as a Δ*Licpa* strain of this parasite was less infective for mammalian hosts and for cells *in vitro*[33].

The synergy between the activities of the CPs that contribute to *L. (L.) mexicana* virulence was verified in assays using parasites that had a combination of CP genes suppressed (*Δcpa/ Δcpb*). These mutants were even less infective in BALB/c mice than other strains with just one type of CP gene suppressed, indicating the complementary activity of these enzymes [34]. A reduction in the virulence of *L.* (*L.*) *mexicana* after CP gene suppression was also observed in infection assays in hamsters and in a human mononuclear phagocytic system [87].

An interesting biological function related to the CPA gene has been described. Double = Δ*cpa/cpb* mutants of *L.* (*L.*) *mexicana* have a disrupted autophagy pathway and are also unable to undergo metacyclogenesis and transformation to amastigotes [77]. CPA and CPB are two major lysosomal cysteine peptidases that may function similarly to the aspartic peptidase PEP4 and the serine peptidase PRB1 in *Saccharomyces cerevisiae*. Autophagy is believed to be necessary to the process of cell differentiation [88].

### *Endogenous Inhibitors* of CPs

The role of the endogenous CP inhibitors expressed by *Leishmania* (ICPs) in immune modulation has also been studied. The *L.* (*L.*) *mexicana* mutants that overexpressed the ICPs led to decreased antibody and IL-4 production but induced the production of increased levels of IFN-γ in murine hosts when compared to a wild-type strain [46]. These observations reinforce the important role of CPs as immune modulation tools, especially in species of the *L.* (*L.*) *mexicana* complex.

### Metalloproteinases of Leishmania as virulence factors

The major surface protein (MSP or gp63) is a metalloproteinase (MP) that belongs to the metzincin class (peptidase family M8) and is abundantly expressed on the surface of *Leishmania* spp. and other related trypanosomatid protozoans [89-92]. Its biological activity is associated with protecting the parasites against the action of host enzymes in the midgut of insect vectors and the phagolysosomes of macrophages. Additionally, gp63 is required for the resistance of promastigotes to complement-mediated lysis in the mammalian host, as the presence of an enzymatically active form of this proteinase greatly reduced the fixation of the terminal complement components on parasites and increased the conversion of C3b to the inactive form C3bi [53,54]. The cellular localization of metalloproteinases with domains homologous to gp63 was investigated in *L.* (*V.*) *braziliensis* and showed that these enzymes are mainly located in the flagellar pocket of the parasite [93]. Studies of the direct effects of metalloproteinases on the immune system of mammalian hosts show that gp63 is important during macrophage infection and modulates the cytokine immune response, as downregulation of gp63 expression in parasites rendered them more susceptible to complement-mediated lysis and led to the development of a Th1-type response at the site of inoculation and its draining lymph node [55].

Natural killer (NK) cells are another type of immune cell that have been shown to be affected by *Leishmania* gp63. The ability of promastigotes to suppress this cell type is dependent on gp63 expression, as a *L.* (*L.*) *major* gp63 mutant strain loses its ability to hinder the proliferation of NK cells and to inhibit the expression of surface receptors on these cells [57].

Gp63 has also been shown to interfere with signaling cascades and to affect transcription factors, thus preventing host cells from adequately responding to the parasites during infection. One example of such activity is the gp63-dependent cleavage of c-Jun, the central component of the transcriptional complex AP-1, by *L.* (*L.) mexicana* parasites. Interestingly, gp63 retrieved from culture supernatants maintains the capacity to cleave c-Jun, suggesting that when secreted by the parasites, this enzyme may use a phagocytosis-independent mechanism to enter host cells [59].

An alternative immune modulation effect of gp63 occurs by the activation of protein tyrosine phosphatases (PTPs) in macrophages, leading to decreased NO production and attenuated innate inflammatory responses, therefore increasing the parasite’s odds of survival. It was reported that gp63 activates at least three macrophage PTPs through its proteolytic activity and that this process is partially dependent on a lipid raft-based mechanism [94].

Similar to CPB, g63 has also been implicated in the cleavage of NF-κB, but this activity has a more subtle and specific purpose than simply preventing the transcriptional factor from reaching the nucleus. Instead, gp63 cleaves the NF-κB subunit p65 into a smaller subunit (p35) that enters the nucleus of the host cell and triggers the expression of chemokines. By subverting the proper function of the transcriptional machinery, the parasites are able to recruit phagocytic cells to serve as hosts while preventing the expression of host factors as IL-12 and iNOS that threaten their survival, [56]. The gp63 from *L. (L.) major* and *L. (L.) donovani* can also cleave CD4 glycoprotein on human T cells in culture, revealing yet another example of the influence that *Leishmania* parasites have on the host immune system. The cleavage was measured by assessing the binding of specific antibodies binding CD4 glycoprotein on the surface of cells, and this effect was detected using both promastigotes and purified gp63 on the cell cultures [58].

### Serine-proteinases of *Leishmania* as virulence factors

Serine proteinases (SPs) have also been shown to act as virulence factors and influence host immune responses during *Leishmania* infection, but, unlike CPB for cysteine-proteinases and gp63 for metalloproteinases, there is not yet a specific SP that has been shown to be responsible for these effects. Reports indicate that the levels of surface SPs diminishes in attenuated strains of *L* (*L.*) *donovani* and that a 115 kDa SP seems to be related to the parasite’s ability to infect the host [95].

A SP of *Leishmania*, oligopeptidase B (OPB; Clan SC, family S9A oligopeptidase B), was identified and characterized by mass spectrometry and gene deletion [60]. It was suggested that during differentiation to the amastigote form, OPB is upregulated and participates in the covering of parasite surface with enolase and plasminogen. The amastigotes are then able to replicate undetected within the macrophage.

The direct effects of OPB on the host immune system was demonstrated by examining the effect of infection with an OPB mutant strain on the expression of host genes. Infection of macrophages in culture with a wild-type *L.* (*L.*) *donovani* strain results in changes in the expression of 23 genes, but infection of these same cells with a mutant strain in which the oligopeptidase B gene was deleted leads to changes in 495 genes, an increase of approximately 21x in the number of genes affected by the infection. This suggests a relationship between oligopeptidase B expression and the ability of *Leishmania* to remain undetected in macrophage infections [60]. OPB-deficient *L.* (*L.*) *major* mutants were recently shown to be able to grow normally as promastigotes even though they are slightly deficient in their ability to undergo differentiation to metacyclic promastigotes, but they were significantly less able to infect and survive within macrophages *in vitro* despite maintaining their virulence in mice. These data suggest that *L.* (*L.*) *major* OPB itself is not a prevalent virulence factor but rather acts in conjunction with other factors, indicating functional differences between trypanosomes and *Leishmania* in their interaction with the mammalian host [96].

Another class of SP, the subtilisin protease (SUB; Clan SB, family S8) of *L. (L.) donovani,* was cloned and shown to possess a unique catalytic triad. When this SP gene was deleted, the ability of the parasite to undergo promastigote to amastigote differentiation *in vitro* was reduced. Furthermore, the activity of this *Leishmania* SP is increased by several fold in amastigotes when compared to promastigotes, suggesting an important role for this enzyme in the vertebrate-inhabiting stages of the parasite [61].

Additional evidence of immune modulation by SPs was obtained by the observation that immunization with a soluble proteases fraction isolated from a mixture of *L.* (*L.*) *amazonensis* antigens increased the susceptibility of mice to a subsequent experimental infection. This effect was eliminated by treating the protease fraction with SP inhibitors but not with CP inhibitors [62].

### Importance of proteinases from mammalian host in the progression of the infection

Although the proteinases from parasites play important roles in immunomodulating the host response and, consequently, the outcome of the infection, proteinases from the host also affect the dynamics of the infection and the development of the lesion. For example, matrix metalloprotease-9 (MMP-9) interferes with the re-epithelization of chronic wounds in humans. In situations where the inflammation continues for a long period, TNF-α and, subsequently, MMP-9 persist, thereby preventing the migrating keratinocytes from forming new attachments to a newly synthesized basement membrane. This suggests a mechanism whereby the presence of high levels of MMP-9 delays the process of normal wound healing [97].

These data are correlated with findings that associate a high number of cells producing IFN-γ, IL-10 and TNF-α, in addition to elevated levels of MMP-9 activity in lesions, with a poor response to therapeutics for cutaneous leishmaniasis (CL) [98]. Indeed, infection with *L.* (*L.*) *chagasi* stimulates murine macrophages to produce MMP-9 [99]. Conversely, elevated levels of MMP-2 mRNA were detected in lesions from patients with good responses to treatment [98], an observation consistent with other reports that showed that increased MMP-2 levels were required for cutaneous wound re-epithelization [100]. Interleukin-10 is observed at higher levels in patients with good immune responses [98] and seems to be unique among the cytokines in its ability to suppress the production and activation of MMPs, thus having an important matrix-protective role during inflammation [101].

### Remarks

The network of interactions that take place during the evolution of the *Leishmania* infection in the mammal host is highly complex and involves a series of responses and counter-responses from both organisms. In this context, proteinases appear as factors of pivotal importance, playing central roles in many of the interactions between parasite and host (Table 1).

Although most of the literature currently available on this subject is focused on cysteine-, metallo- and serine-proteinases from *Leishmania* species, the importance of host proteinases, such as matrix metalloproteinases, and their role in the subversion of host’s immune responses by the parasites must not be overlooked, as some reports already point to their direct contribution to determining the outcome of infections. Furthermore, it is necessary to analyze the participation of aspartyl-proteinases from *Leishmania* species, as their expression changes between morphological forms and may therefore be related to the responses necessary to survive in distinct micro-environments [65]. Therefore, to fully understand how the *Leishmania* infection progresses and to be able to find suitable targets for drug development and options for patient treatment, it is important to consider the influence of proteinases from host and parasite alike, as disregarding one in favor of the other may lead to incorrect conclusions and inadequate, if not harmful, treatment of the disease.

## Competing interests

The authors declare that they have no competing interests.

## Authors’ contributions

CRA formulated the idea and CRA, MSA, BASP and MLRG wrote the manuscript. All authors approved the final version of this manuscript.
